# Inhibition of Autoimmune Diabetes in NOD Mice by miRNA Therapy

**DOI:** 10.1371/journal.pone.0145179

**Published:** 2015-12-16

**Authors:** Duncheng Wang, Iryna Shanina, Wendy M. Toyofuku, Marc S. Horwitz, Mark D. Scott

**Affiliations:** 1 Centre for Innovation, Canadian Blood Services, Ottawa, Ontario, Canada; 2 Centre for Blood Research, University of British Columbia, Vancouver, British Columbia, Canada; 3 Department of Microbiology and Immunology, University of British Columbia, Vancouver, British Columbia, Canada; 4 Department of Pathology and Laboratory Medicine, University of British Columbia, Vancouver, British Columbia, Canada; University of Cincinnati College of Medicine, UNITED STATES

## Abstract

Autoimmune destruction of the pancreatic islets in Type 1 diabetes is mediated by both increased proinflammatory (Teff) and decreased regulatory (Treg) T lymphocytes resulting in a significant decrease in the Treg:Teff ratio. The non-obese diabetic (NOD) mouse is an excellent *in vivo* model for testing potential therapeutics for attenuating the decrease in the Treg:Teff ratio and inhibiting disease pathogenesis. Here we show for the first time that a bioreactor manufactured therapeutic consisting of a complex of miRNA species (denoted as TA1) can effectively reset the NOD immune system from a proinflammatory to a tolerogenic state thus preventing or delaying autoimmune diabetes. Treatment of NOD mice with TA1 resulted in a systemic broad-spectrum upregulation of tolerogenic T cell subsets with a parallel downregulation of Teff subsets yielding a dramatic increase in the Treg:Teff ratio. Moreover, the murine-derived TA1 was highly effective in the inhibition of allorecognition of HLA-disparate human PBMC. TA1 demonstrated dose-responsiveness and exhibited equivalent or better inhibition of allorecognition driven proliferation than etanercept (a soluble TNF receptor). These findings demonstrate that miRNA-based therapeutics can effectively attenuate or arrest autoimmune disease processes and may be of significant utility in a broad range of autoimmune diseases including Type 1 diabetes.

## Introduction

Autoimmune destruction of pancreatic islets in Type 1 Diabetes (T1D) occurs via T-lymphocyte (T cell) dependent pathways.[1–3] Elucidation of the role of T cells in T1D has been most effectively examined in the non-obese diabetic (NOD) mouse model. In the NOD mouse, evidence suggests that a deficit in regulatory T cell (Treg) control over diabetogenic T effector (Teff) cells leads to the development of insulitis and disease.[2–12] Indeed, changes in the Treg:Teff ratio (*i*.*e*., balance) occurring at 3–4 weeks of age initiates disease progression.[2] Consistent with the murine findings, human studies have demonstrated that T1D Tregs exhibit an impaired ability to suppress Teffs.[13] Thus, the emergence of an aggressive diabetogenic lymphocyte response in NOD mice, and likely humans, is dependent upon a change in the Treg:Teff ratio.

Consequent to the central role of the T cell in the pathogenesis of diabetes, pharmacological approaches for the prevention/inhibition of T1D have primarily targeted T cells. Importantly, whether via cytotoxic targeting, Treg expansion, cytokine adsorption and/or supplementation, monoclonal antibodies, or amelioration of redox-injury, a consistent therapeutic goal has been to ultimately change the Treg:Teff ratio.[1,14–19] However, these approaches are characterized by limited windows of therapeutic efficacy, short duration of efficacy and/or significant toxicity. Hence, the development of novel, immunomodulatory therapeutics directly altering T cell subset differentiation and, ultimately the Treg:Teff ratio, would be of significant clinical utility in T1D.

Recent studies have demonstrated that microRNAs (miRNA) are key regulators of cellular processes including the immune response.[20,21] miRNA are short (~22 nucleotides) single-stranded RNA molecules found in all eukaryotes. It is estimated that ~60% of mammalian genes are targeted by one or more miRNA.[22,23] Indicative of their evolutionary and functional importance in gene regulation, miRNA are highly conserved between species (*e*.*g*., mouse and human).[22] While miRNA are most commonly found intracellularly, significant levels of stable miRNA are also found in the serum of mammals suggesting an important messenger/regulatory role. Over the last few years, the role of miRNA in disease processes has become an active research area and recent findings suggest that they may be biomarkers, or possibly mediators, of T1D.[24–26]

However, miRNA regulation of protein expression is complex and the role of a single miRNA is difficult to assess as it can potentially affect hundreds of genes and, conversely, individual genes can be regulated by multiple miRNA.[23] Consequent to this regulatory complexity, most studies have focused on miRNA as disease biomarkers, not as therapeutic agents.[27–30] From a bioregulatory approach, it is more probable that the differential expression of multiple miRNA are necessary for initiating a biological response. Hence, it is this *‘pattern of miRNA expression’* (encompassing increased, decreased and static levels of multiple miRNAs) that must be mimicked to achieve pharmacologically effective miRNA-based therapeutics. However, successful implementation of this bioregulatory approach has not previously been possible.

Surprisingly, cellular bioengineering via polymer grafting may yield an unexpected avenue by which a miRNA bioregulatory approach can be achieved. We previously demonstrated that allorecognition of HLA/MHC-disparate donor leukocytes could be abrogated by the covalent grafting of methoxypolyethylene glycol (mPEG) to the membrane of cells.[31–38] Moreover, the immunocamouflage of allogeneic cells by the grafted polymer yielded a non-proliferative tolerogenic/anergic environment, both *in vitro* and *in vivo*, that was mediated by soluble factors.[33,34,36] It was initially hypothesized that altered cytokine expression patterns, characterized by upregulation of tolerogenic (*e*.*g*., IL-10) and downregulation of proinflammatory (*e*.*g*., IL-2, IFN-γ, IL-17A, TNF-α, and Il-6) cytokines, mediated this effect.[36] Intriguingly, the therapeutic component was found to reside almost solely within the miRNA fraction of conditioned media or plasma. Using a bioreactor approach, a purified miRNA-based cocktail (denoted as Tolerance Agent 1; TA1) has been reproducibly generated. To experimentally assess whether TA1 could arrest or delay a T cell-dependent autoimmune disease process, the NOD mouse model of T1D was examined. NOD mice were treated with saline or TA1 and examined for T cell subset differentiation, Treg:Teff balance, diabetes incidence, and pancreatic islet morphology.

## Materials and Methods

### Bioreactor production of the TA1 miRNA

Production of the TA1 miRNA-based biologic was accomplished using an *in vivo* murine (Balb/c, H-2^d^; and C57Bl/6, H-2^b^) bioreactor system. Mice were transfused (*i*.*v*.) with saline (control) or mPEG-grafted (20 kDa SCmPEG; 2 mM grafting concentration per 4 x 10^6^ cells/mL) allogeneic splenocytes as described by Wang *et al*.[36] At 5 days post transfusion, heparinized whole blood was harvested and centrifuged (1,500*g* for 10 minutes; 22°C) for collection of the control or ‘conditioned’ plasma. Naïve and TA1-miRNA was extracted from the control and conditioned plasma, respectively, using the mirVana^TM^ PARIS^TM^ kit (Cat. No. AM1556, Ambion, Life Technologies; Grand Island, NY). Following processing, the highly enriched small RNA fraction containing miRNA was prepared using RNase/DNase free isotonic saline. To confirm that miRNA was the active component, aliquots of conditioned plasma and TA1 were RNase-treated (50 ng RNase A; 10 minutes at 37°C; Life Technologies) to degrade the nucleic acid. The *in vivo* immunomodulatory effects of the naïve-plasma, naïve-miRNA, conditioned-plasma and TA1-miRNA (all ± RNase A treatment) were confirmed *in vivo* using immunocompetent Balb/c or C57Bl/6 mice or *in vitro* using human or murine mixed lymphocyte reactions. To partially characterize and quantify the relative abundance the miRNA species present in the TA1 bioreactor produced preparation, qPCR miRNA microarrays were analyzed using an Applied Biosystems StepOnePlus^TM^ Real Time PCR System (ThermoFisher Scientific, Grand Island, NY) and the Mouse Immunopathology miScript miRNA PCR Array (Qiagen, Frederick, MD). The PCR array profiles the expression of 84 miRNA differentially expressed during normal and pathological immune responses. It is worth noting that the 84 miRNA examined are not all inclusive and that other miRNA are likely present and could be of immunoregulatory importance. The data shown represent a minimum of 3 biological replicates analyzed independently by qPCR.

### NOD mouse study

All murine studies were performed following protocol approval by the University of British Columbia Animal Care Committee and were done in accordance with the Canadian Council of Animal Care guidelines. NOD/ShiLtJ mice were obtained from The Jackson Laboratory (Bar Harbor, ME, USA). To assess the immunomodulatory efficacy of TA1 in autoimmune T1D, age of onset, incidence, T cell differentiation and pancreatic islet morphology were measured in naïve female NOD mice treated at 7 weeks of age with either saline or TA1 (N≥15 per group). Importantly, at 7 weeks of age, the underlying disease process is well underway.[2] Treatment consisted of 3 intravenous *(i*.*v*.*)* injections (100 μl each) at 2 day intervals of isotonic saline or TA1. No further interventions were done. Blood and/or urine glucose levels were determined weekly (One Touch Ultra Glucometer, LifeScan) to indirectly monitor pancreatic (ß cell) function. NOD mice that had two consecutive blood glucose values of ≥ 20 mM were defined as diabetic.

Upon loss of normoglycemia, or at week 30, mice were sacrificed and the spleen, pancreatic lymph node, brachial lymph node and peripheral blood were collected as previously described.[36] Mouse spleens, pancreatic and brachial lymph nodes were dissected and placed into cold phosphate buffered saline (PBS; 1.9 mM NaH_2_PO_4_, 8.1 mM Na_2_HPO_4_, and 154 mM NaCl, pH 7.3) containing 0.2% bovine serum albumin (BSA; Sigma Aldrich, St. Louis, MO) for production of single cell suspensions suitable for flow cytometric analysis. Residual erythrocytes in the splenocytes were removed using BD Pharm Lyse™ lysing buffer (BD Biosciences, San Diego, CA).[36] Pancreatic ß-cell mass and histopathology was determined via quantitative image analysis following insulin immunostaining.[39] Insulitis was scored as normal (0), peri-insulitis (+, ++) and overt insulitis (+++, ++++).

### T cell immunophenotyping by flow cytometry

Samples were acquired and analyzed using a FACSCalibur flow cytometer and CellQuest Pro software (BD Biosciences, San Jose, CA). All antibodies used were obtained from BD-Pharmingen (San Diego, CA) and used per manufacture’s specifications. To best reflect the *in situ* leukocyte subpopulations mediating the autoimmune disease pathology, no exogenous stimulation of the isolated cells was done prior to flow cytometric analysis. Consequent to this decision, subpopulations are somewhat reduced relative to studies in which *in vitro* stimulation (e.g., anti-CD3/CD28) is done prior to flow cytometric phenotyping. Cells (1 x 10^6^ cells total) were washed and resuspended in PBS (0.1% BSA) for flow acquisition. T cells were defined as CD3^+^CD4^+^. Tolerogenic cells populations included: Foxp3^+^ (Treg), IL-4^+^, IL-10^+^, TGF-β^+^ T cells, and CD11c^+^ dendritic cells (DC). In some experiments, the effect of TA1 on CD25^+^ and CD69^+^ regulatory T cells were also determined.[40,41] Teff cell subpopulations included: IL-2^+^; IFN-γ^+^; TNF-β^+^; CD40^+^ and IL-17A^+^ (Th17 cells) populations. For experimental analysis, the Treg:Teff ratio was defined as the ratio of Foxp3^+^ to IL-17A^+^ (*i*.*e*., Th17 cells) cell based on preliminary experiments and the data observed within the study. Hence, Foxp3^+^ and IL-17A^+^ (Th17) cells were simultaneously determined using the BD Th17/Treg kit (BD Pharmingen, San Diego, CA). All other T cell subset markers were determined independently. All tissues were analyzed on a per mouse basis.

### Human mixed lymphocyte reactions

All experiments using human blood cells were done following protocol approval by the University of British Columbia Clinical Research Ethics Board and in accordance with the Declaration of Helsinki. Following informed written consent, donor whole blood was collected in heparinized Vacutainer^®^ blood tubes (BD, Franklin Lakes, NJ). Peripheral blood mononuclear cells (PBMC) were prepared using Ficoll-Paque PREMIUM (GE Healthcare Bio-Sciences Corp, Piscataway, NJ) and two-way mixed lymphocyte reactions (MLR) were done as previously described.[36] Sample wells were treated with either saline (200 μl) or increasing amounts of TA1 (0–200 μl). Cell proliferation was assessed via flow cytometry using the CellTrace CFSE (Carboxyfluorescein diacetate, succinimidyl ester; 2.5 μM CFSE per 2 x 10^6^ PBMC) Cell Proliferation Kit (Invitrogen by Life Technologies–Molecular Probes, Carlsbad, CA).

### Statistical Analysis

Data analysis was conducted using SPSS (v12) statistical software (Statistical Products and Services Solutions, Chicago, IL, USA). For significance, a minimum *p* value of <0.05 was used. When only two means were compared, student-t tests were performed while one-way analysis of variance (ANOVA) was performed for comparison of three or more means. When significant differences were found, a post-hoc Tukey test was used for pair-wise comparison of means.

## Results

### TA1 immunomodulation—miRNA dependency

**Fig 1:**
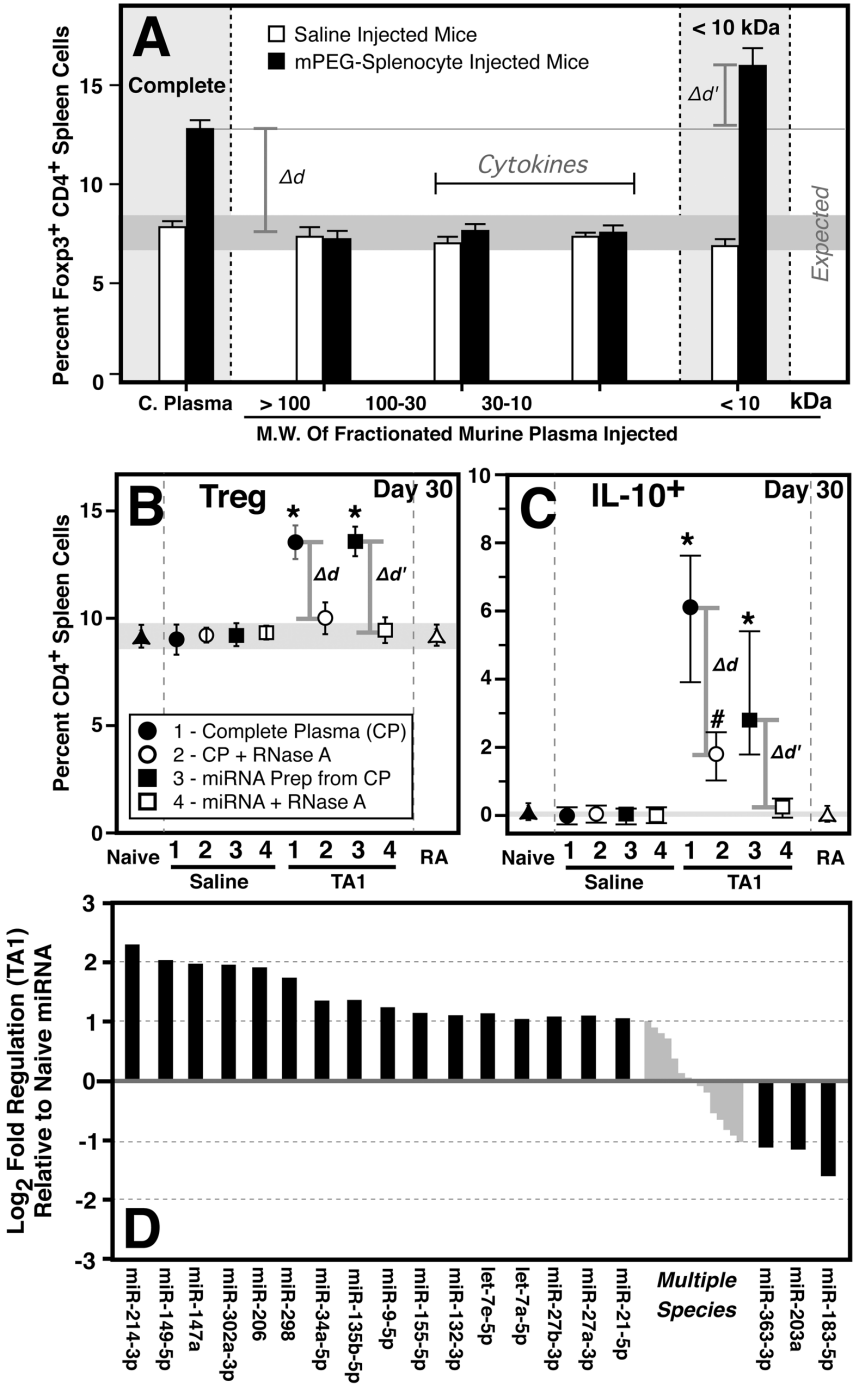
Characterization of miRNA as the functional immunomodulatory agent. **Panel A:** Size fractionation of plasma obtained from saline (white) and tolerized (black) mice was done to isolate the tolerogenic fraction(s). As shown, for fractions >10 kDa, *in vivo* immunodulatory activity was lost (*Δd*) relative to the complete plasma. In contrast, the <10kDa fraction demonstrated significantly elevated Treg levels (*Δd'*; p<0.01) relative to complete plasma. Normal Treg levels for naïve mice are denoted by the shaded grey bar. As shown, saline treatment had no immunomodulatory effect. Splenic Treg cells were assessed 5 days post treatment (N≥5 per group). **Panels B-C:** Comparative *in vivo* effects of plasma or miRNA prepared from saline and tolerized mice on Foxp3^+^CD4^+^ (**B**) and IL-10^+^CD4^+^ (**C**) lymphocytes at 30 days post treatment. Neither complete plasma nor miRNA prepared from saline treated animals exhibited immunomodulatory activity. In contrast, conditioned plasma and miRNA (TA1) prepared from it, exhibited significant (p<0.001) upregulation of both Treg and IL-10+ cells which was lost if the plasma or TA1 (**Panels B-C**; Δd and Δd’, respectively) was treated with RNase A (RA) prior to administration. Direct *in vivo* administration of RA to naive mice had no effect. Values shown are the mean and range of ≥5 animals per group. **Panel D:** Partial characterization of the tolerogenic TA1-miRNA pattern. miRNA shown represent species with significant variance from the non-tolerogenic (naïve) miRNA preparation (0 value; grey line). The tolerogenic pattern consists of miRNA that are both increased and decreased in relative abundance. Note that multiple other miRNA species are relatively unchanged from naive miRNA but may also be of bioregulatory importance in TA1 activity. Data shown represent a minimum of 3 biological replicates analyzed independently by qPCR.

The tolerogenic environment induced by the polymer-grafted leukocytes was previously hypothesized to have arisen from differential cytokine expression.[36] However, as shown in **Fig 1A**, fractionation of conditioned murine plasma demonstrated that the active tolerogenic component did not reside in the cytokine-rich fractions (10–100 kDa) but was solely in the ≤ 10 kDa fraction; *i*.*e*., the miRNA fraction. Indeed, as shown, the cytokine-rich fractions did not confer any immunomodulatory effects *in vivo* in immunocompetent Balb/c mice at 5 days post treatment. Moreover, the tolerogenic effects of both the tolerized plasma and the purified TA1-miRNA were persistent to at least 30 days post-TA1 treatment as evidenced by the maintenance of elevated Treg and IL-10^+^ lymphocyte populations (**Fig 1B and 1C**). In stark contrast, neither plasma obtained from naive mice, nor miRNA purified from naive plasma, exerted any immunomodulatory effects on Treg or IL-10^+^ T cell populations. To determine whether miRNA (or possibly other small RNAs) were the immunomodulatory active component, tolerized plasma and TA1 were treated with RNase A. As shown in **Fig 1B and 1C**, treatment of conditioned plasma and TA1 (Δd and Δd’, respectively) with RNase A significantly decreased or abolishment their *in vivo* effects. Conversely, RNase A treatment of naive plasma and the naïve miRNA preparation (nor direct *i*.*v*. injection of RNase A) had no *in vitro* or *in vivo effect* on T cell differentiation. The pattern of TA1-associated miRNA expression was determined by qPCR (**Fig 1D). The** putative functions of the identified miRNA (as currently described in the literature) are provided in the **S1 Table**. As noted the TA1-mediated immunomodulatory effect arises from a complex patter of miRNA expression encompassing increased, decreased and static levels of multiple miRNA species. This likely reflects the complex miRNA bioregulatory mechanism necessary to regulate protein expression, cell proliferation and differentiation. It is important to note that, while several species are individually identified, multiple other miRNA are present (denoted by grey bars) that are marginally changed relative to naive miRNAs but may still be of significant bioregulatory importance in mediating TA1 activity.

**Fig 2:**
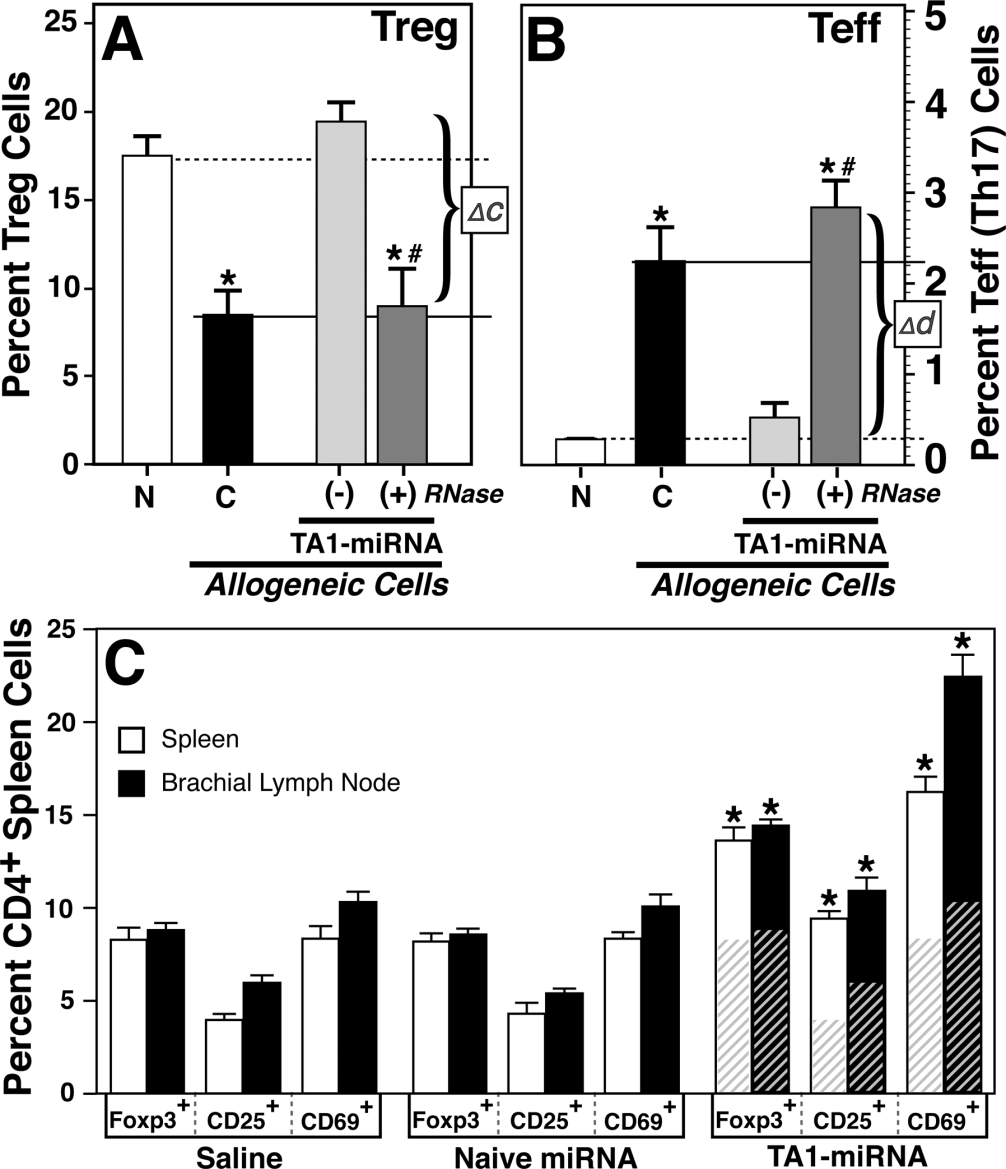
TA1 results in a decreased *in vivo* inflammatory response upon allogeneic cells challenge. In immunologically competent mice (Balb/c), TA1 completely abrogates the inflammatory response to allogeneic (C57Bl/6) splenocytes. Shown are the levels of Treg and Teff (Th17) cell levels in the spleen of naïve (N), allogeneic challenged (C) and TA1 treated (± RNase A treatment) mice 5 days post treatment. TA1 was administered 24 hours prior to administration of the allogeneic splenocytes. As noted, the ‘active’ agent of the TA1 preparation on Treg and Teff levels was fully degraded by RNase treatment (*Δc/d*, respectively). Dashed lines represents naïve resting levels of Treg or Th17 cells while the solid lines denote the mean Treg and Th17 cells of control mice 5 days post transfusion of unmodified, viable, allogeneic splenocytes. Data shown is the mean ± SD of a minimum of 8 mice per group. * Denotes significantly different (p<0.001) from naïve mice. **#** Denotes significantly different (p<0.001) from TA1 treated mice. **Panel C:** *In vivo* effects of saline, naive miRNA or TA1-miRNA on Foxp3^+^, CD25^+^ and CD69^+^ T cells in the spleen and brachial lymph node. For ease of comparison, the cross-hatched regions of the TA1 group represent the mean saline values. Cell populations were determined 5 days post treatment with a minimum of 5 animals per treatment group. (*) Indicates significantly (p<0.001) different from saline group in Panels A-B. In Panel C (#) denotes significantly reduced from sample 1 but still significantly (p<0.01) elevated relative to saline group.

To assess whether TA1 could effectively blunt an 'autoimmune-like' inflammatory event *in vivo*, immunologically normal mice were challenged with H-2 disparate allogenic cells. As shown in **Fig 2**, transfusion of naïve Balb/c mice with C57Bl/6 splenocytes resulted in a significant (>50%; p <0.001) decrease in Tregs with a corresponding increase in Th17 (~10-fold; p<0.001) cells at 5 days post challenge. However, TA1 pretreatment (5 days) prior to allogenic splenocytes challenge prevented both the downregulation of Foxp3^+^ Treg (**Fig 2A**) and the upregulation of Th17 (**Fig 2B**) cells thus maintaining a Treg:Teff ratio characteristic of naïve mice. Importantly, the immunomodulatory effects of exogenous TA1 on Treg and Teff subsets was completely abolished by pre-treatment of TA1 with RNase A (Δc and Δd, respectively). Moreover, the effect of TA1 on regulatory cells in immunocompetent animals was not limited to Foxp3^+^ Tregs. As shown in **Fig 2C**, TA1 induces significant (p<0.001) increases in both CD25^+^ and CD69^+^ T cells. While CD25^+^ are of obvious importance as regulatory T cells, the CD69^+^ cells may be of equal or greater importance as recent studies have demonstrated their role in the maintenance of immune tolerance by Foxp3^+^ regulatory T cells.[40,41] While not shown directly, the Foxp3^+^ cells were typically both CD69^+^ and CD279^+^ (PD1).

### NOD T1D age of onset, incidence and islet histology

**Fig 3:**
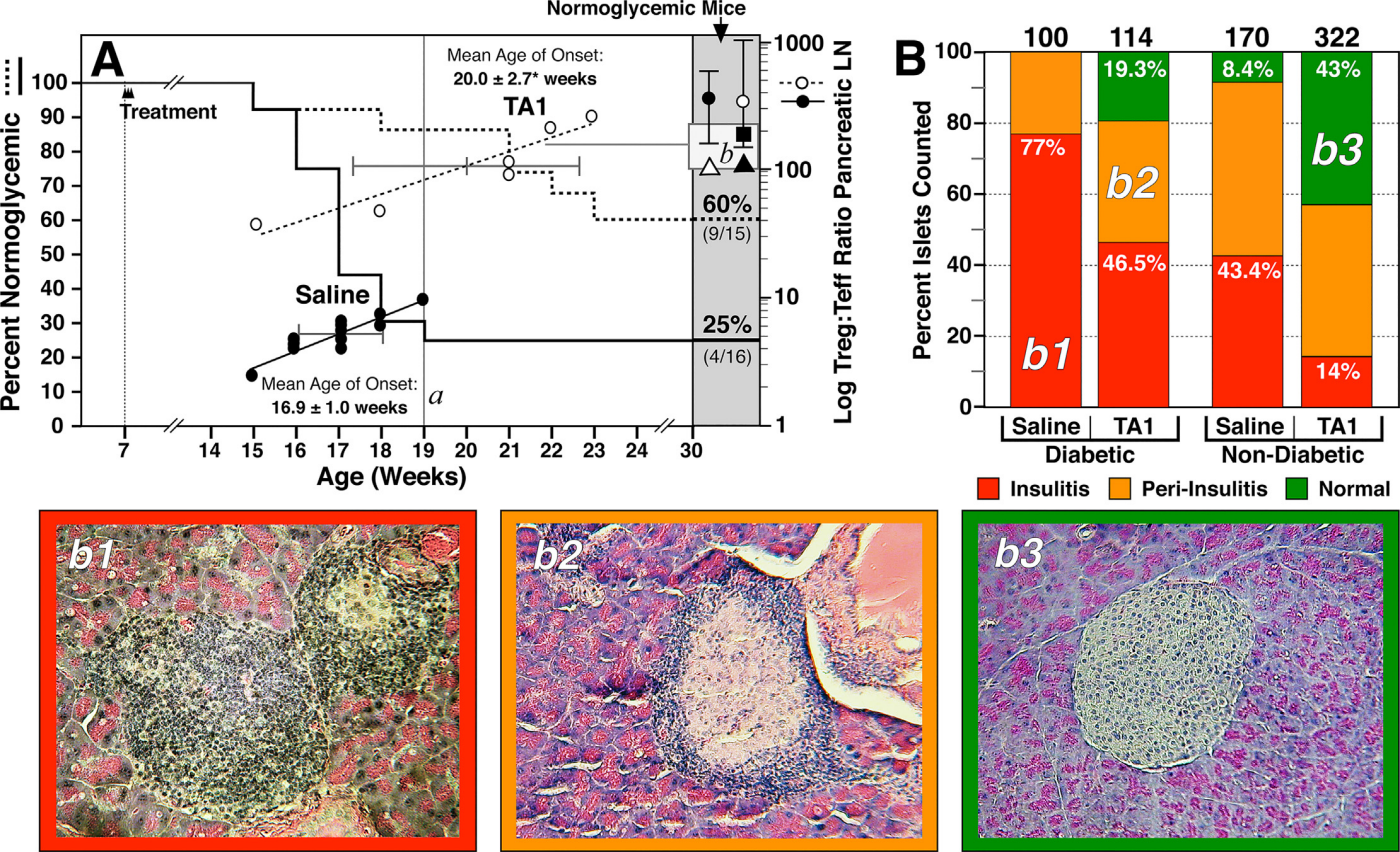
TA1 administration prevents (p≤0.01) or delays T1D (p≤0.01) progression in NOD mice, alters the ratio of Treg:Teff cells and improves islet histology. **Panel A:** Mice were treated (*i*.*v*.) with either saline or TA1 at 7 weeks of age and blood glucose was measured to a maximum of 30 weeks. Shown are the percentage of animals remaining normoglycemic (*left axis*). As shown, only 25% of control mice were normoglycemic at week 19 (line *a*) while 87% of TA1 remained normal. At 30 weeks, 25% of untreated mice and 60% of TA1-treated mice remained normoglycemic. Conversion to T1D correlated in both groups with the log Treg:Teff ratio (*left axis*). TA1 significantly increased the Treg:Teff ratio in all animals (Diabetic and non-diabetic). The Treg:Teff ratio of normoglycemic saline (● mean 286; range 170–680) and TA1 (○ mean 255; range 140–1040) treated mice (shade box; right) were similar but significantly (p<0.001) higher than diabetic mice. Analysis of the Treg:Teff ratio of diabetic and non-diabetic saline and TA1 mice suggests that an *inexact* threshold level (box *b*) in the Treg:Teff ratio may exist for protection against progression to T1D. Also shown are the Treg:Teff ratios for immunocompetent C57Bl/6 and Balb/c mice (Δ, 91.5; ■, 198; respectively) and the asymptomatic 7 week old NOD mice (▲;103). Diabetic tissues were harvested at time of conversion, non-diabetic tissues were harvested at week 30. Diabetic values are the mean ± SD of 12 saline and 6 TA1 treated NOD mice. For diabetic animals (saline and TA1 treated) the mean age of onset ± SD is shown via both text and horizontal bar. Non-diabetic results are the mean ± SD of 4 saline and 9 TA1 treated NOD mice. **Panel B:** TA1 inhibits pancreatic islet insulitis as demonstrated by the increased number of normal (*b3*) and peri-insulitis (*b2*) islets relative to control NOD mice. Untreated NOD mice exhibited virtually no normal islets (either in diabetic or non-diabetic mice) with a heavy preponderance of overt insulitis (*b1*). The numbers shown at the top of each column represent the number of individual islets graded per condition from a minimum of 5 animals per group.

**Fig 4:**
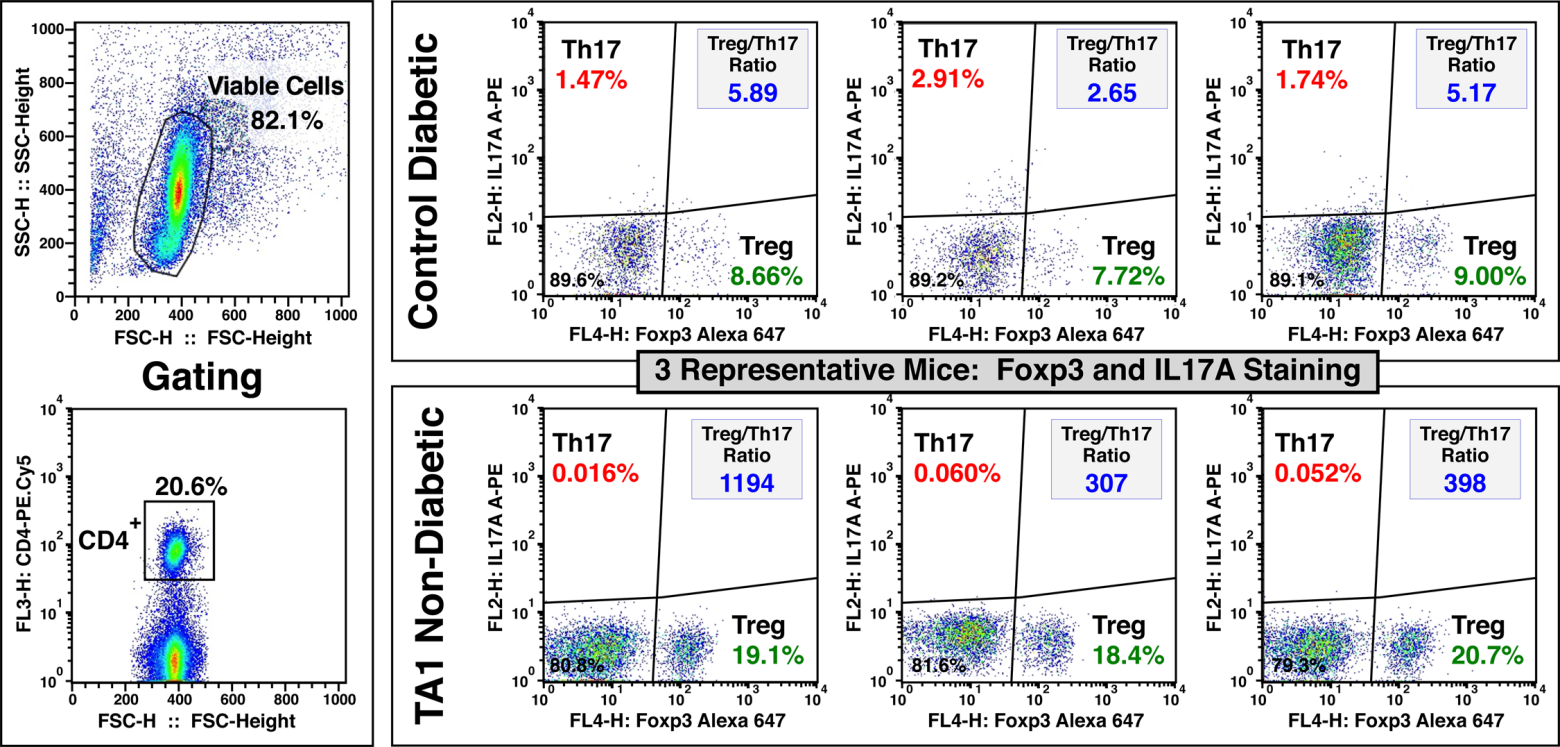
TA1 treatment results in significantly increased Foxp3^+^ (Treg) cells while simultaneously decreasing IL-17A^+^ (Th17) T cells. Shown are the flow cytometric data for 3 representative animals from the Control Diabetic (Total N = 12) and TA1 Non-Diabetic (Total N = 9) groups presented in **Fig 5**. The ratio of Foxp3^+^ to Il-17A^+^ Cells (i.e., the Treg:Teff ratio) is shown in the upper right quadrant. A high Treg:Teff ratio (*i*.*e*., > ~200) correlated with maintenance of normoglycemia (**Fig 3A**). The gating strategy utilized in shown in the left-most panel. To best reflect the *in situ* leukocyte subpopulations mediating the autoimmune disease pathology, no exogenous stimulation of the isolated cells was done prior to flow cytometric analysis.

To experimentally determine if TA1 could be used therapeutically in an autoimmune disease, female NOD mice were treated at 7 weeks of age with saline or TA1. Importantly, 7 weeks is well beyond the onset of pancreatic islet immune cell infiltration that begins at 3–4 weeks of age.[2] As shown in **Fig 3A**, saline treated NOD mice exhibited a rapid onset of diabetes with 75% (12 of 16) of the mice becoming diabetic by 19. Subsequent to week 19, no additional mice became diabetic. In contrast, by week 19 only 13% (2 of 15) of the TA1 treated mice became diabetic with an additional 4 mice becoming diabetic between weeks 21 and 23 (total diabetic 6/15; 40%). Analysis of the Treg:Teff ratio of the pancreatic lymph node demonstrated that a low ratio was correlated with the early onset of diabetes while an elevated Treg:Teff ratio was associated with either a significantly delayed mean age of onset (20.0 ± 2.7 versus 16.8 ± 1.0 for TA1 and saline treated diabetic mice respectively; p≤0.01) or absence (p≤ 0.01 for TA1 relative to saline treatment) of disease (**Figs 3A and 4**). These findings correlated with a significant change (p<0.001) in the Treg:Teff ratio occurs between the 7 week old asymptomatic NOD mouse (ratio of 103) and the diabetic NOD mouse (mean ratio of 4.7; *i*.*e*., a ~95% reduction) at time of sacrifice (**Fig 4**). Of note, 7 week old NOD mice have a Treg:Teff ratio virtually identical to C57Bl/6 mice (91.5) but roughly half that of naive Balb/c (198).

In comparison to saline treated mice, TA1 treatment universally increased (p<0.001) the Treg:Teff ratio; even in mice that developed T1D. For diabetic TA1-treated mice, an aggregate Treg:Teff ratio of 70 was observed (still lower than at age 7 weeks). Non-diabetic mice in both the saline and TA1 groups demonstrated significantly (p<0.001) elevated Treg:Teff ratios (**Figs 3 and 4**). For non-diabetic saline and TA1-treated mice the Treg:Teff ratio was significantly higher with mean values of 286 (range 170–680) and 255 (range of 140–1040), respectively. These results suggests that the maintenance of normoglycemia in mature NOD mice required a dramatic tolerogenic reorientation of the immune system over that seen in 7 week old asymptomatic NOD or immunologically competent (e.g., C57Bl/6 and Balb/c) mice. Further analysis of the Treg:Teff data observed in diabetic and non-diabetic mice suggests that a threshold effect may exist.

The aggregate effect of the TA1-mediated changes in the Treg:Teff balance was not only reflected by the decreased incidence of diabetes (**Fig 3A**) but also by improved ß-cell islet histology(**Fig 3B**). As demonstrated in **Fig 3B**, untreated NOD mice (independent of diabetes status) exhibited very high levels of insulitis (*b1*) and peri-insulitis (*b2*) with few, if any, normal islets (*b3*). In stark contrast, TA1 treatment significantly improved islet histology. In the TA1 treated, non-diabetic, mice 43% of islets were graded as normal and only 14% showed overt insulitis suggesting that ~86% of islets were capable of some degree of insulin secretion. Perhaps surprisingly, even in the diabetic TA1-treated mice >19% of islets were normal; greater than twice the number observed in the non-diabetic (~8%) control mice. The effect of TA1 on inhibiting islet inflammation, thus preserving islet structure and function, was suggestive of a systemic tolerogenic reset of the NOD mouse lymphoid microenvironment.

### Lymphoid microenvironment

**Fig 5:**
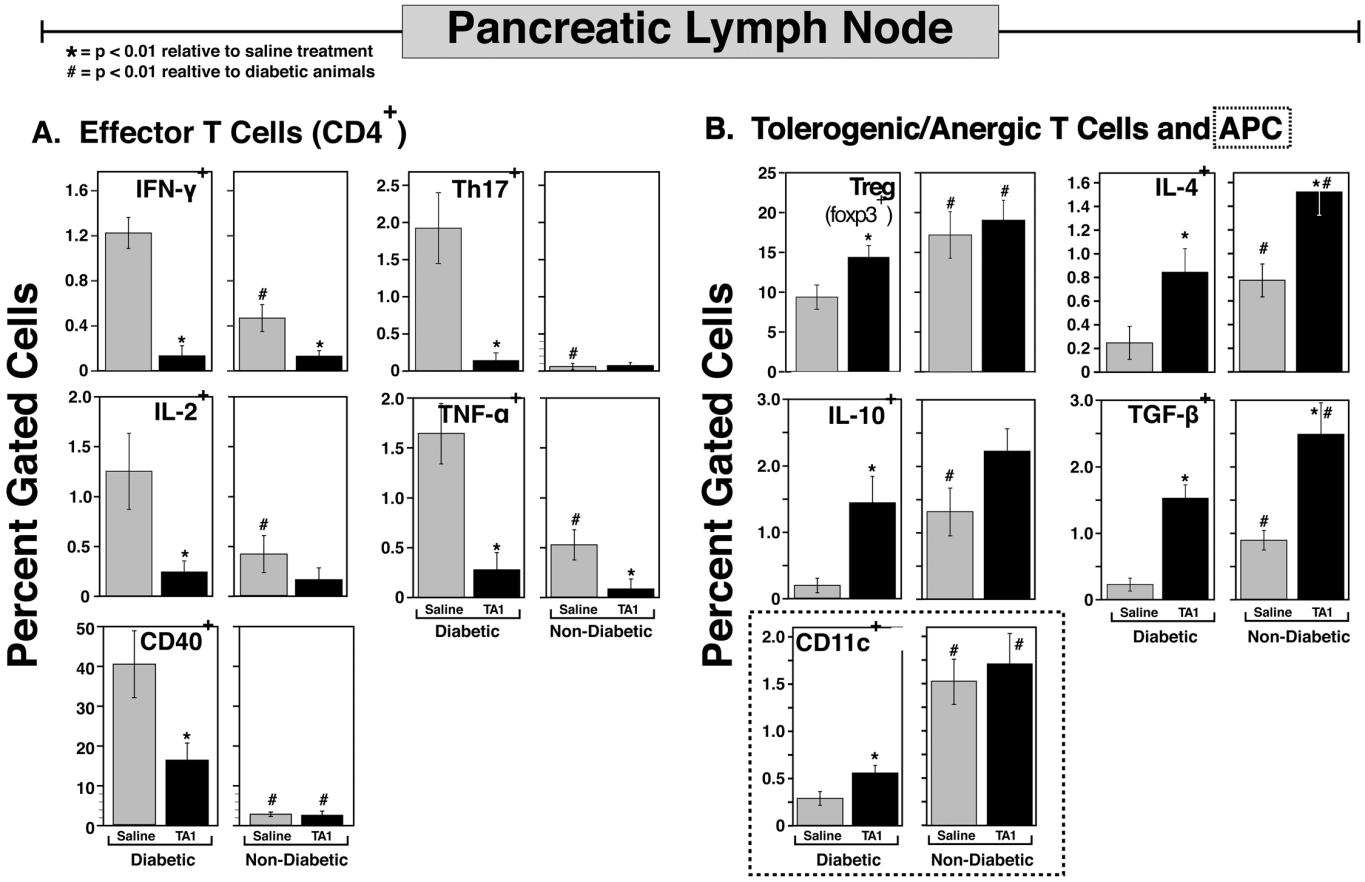
TA1 therapy dramatically alters the expression of multiple pro-inflammatory (A) and tolerogenic/anergic (B) T cell subsets in the pancreatic lymph node. In addition TA1 favored pro-tolergenic changes in DC (CD11c+) cells (B). Diabetic tissues were harvested at time of conversion; non-diabetic tissues were harvested at week 30. Diabetic values are the mean ± SD of 12 saline and 6 TA1 treated NOD mice. Non-diabetic results are the mean ± SD of 4 saline and 9 TA1 treated NOD mice. Representative flow cytometic data are provided in S1 and S2 Figs To best reflect the *in situ* leukocyte subpopulations mediating the autoimmune disease pathology, no exogenous stimulation of the isolated cells was done prior to flow cytometric analysis.

**Fig 6:**
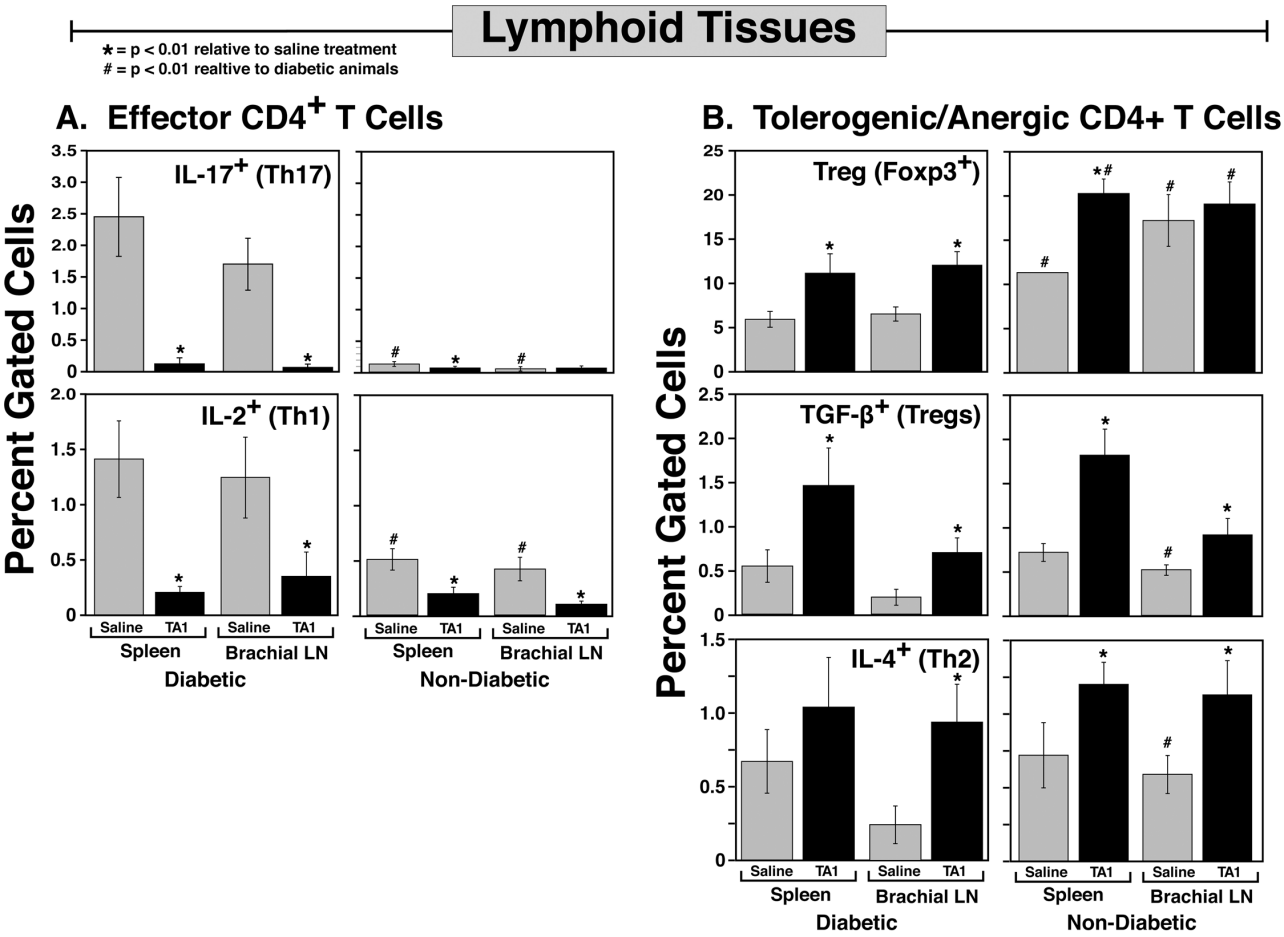
The systemic immunomodulatory effects of TA1 therapy are further evidenced by the tolerogenic skewing of the immune cell subpopulations in the spleen and brachial lymph node. Similar to the pancreatic lymph node, the spleen and brachial lymph node show increased tolerogenic cell populations and decreased proinflammatory cells following TA1 administration at 7 weeks of age. Diabetic tissues were harvested at time of conversion; non-diabetic tissues were harvested at week 30. Diabetic values are the mean ± SD of 12 saline and 6 TA1 treated NOD mice. Non-diabetic results are the mean ± SD of 4 saline and 9 TA1 treated NOD mice. To best reflect the *in situ* leukocyte subpopulations mediating the autoimmune disease pathology, no exogenous stimulation of the isolated cells was done prior to flow cytometric analysis.

The effects of TA1-miRNA therapy on the pancreatic (**Fig 5**) and non-pancreatic (**Fig 6**) lymphoid microenvironments were assessed. As demonstrated in **Fig 5A and S1 Fig**, the progression to diabetes in control NOD mice was characterized by significantly elevated levels of most Teff subsets (IFN-γ^+^, Th17^+^, IL-2^+^, TNF-α^+^ and CD40^+^ lymphocytes) in the pancreatic lymph node. Importantly, to most accurately reflect the *in vivo* pathology of the control (saline) and TA1-treated NOD mice, flow cytometric analysis of the recovered leukocytes were assessed in the absence of any *in vitro* stimulation. Consequent to this decision, subpopulations are somewhat reduced relative to studies in which *in vitro* stimulation (e.g., anti-CD3/CD28) is done prior to flow cytometric phenotyping. As shown, TA1 administration dramatically (p<0.01) blunted the *in vivo* expansion of Teff subsets in the pancreatic lymph node relative to both the diabetic or non-diabetic control NOD mice. Interestingly, with regards to the Teff cell subsets (with the exception of CD40^+^ cells) no significant differences were noted between the diabetic and non-diabetic TA1-treated animals. In large part, this lack of obvious differences in the Teff subset levels between the TA1-diabetic and TA1-non-diabetic groups arose as a consequence of the very low levels of the pro-inflammatory cells noted in *all* of the TA1 treated animals (perhaps arising from the increase in the tolerogenic cell populations; described below).

The lone exception to broad-spectrum TA1-mediated decrease in effector cells was the CD40^+^ subset. While TA1 significantly reduced (by ~60%) CD40^+^ cells in the diabetic NOD mice, the level was still highly elevated relative to both (control and TA1-treated) non-diabetic groups (~17% versus ~2%). Previous studies have shown that anti-CD40L mAbs can block insulitis if administered before 3 weeks of age suggesting a critical role for CD40.[42,43] In light of these studies, it is important to note that TA1 was administered at 7 weeks of age and was still capable of significantly decreasing the CD40^+^ subpopulation. It is possible that additional, or earlier, TA1-treatment would further reduce CD40 expression and substantially reduce the risk of T1D.

Of potentially greater importance, TA1 treatment significantly increased a broad range of tolerogenic/anergic cells present in the pancreatic lymph node (**Fig 5B; S2 Fig**). While Treg (Foxp3^+^CD4^+^) cells were increased by TA1 (**Figs 4 and 5B**), even more dramatic (relative to the saline treated mice) miRNA-mediated increases were noted in IL-4^+^, TGF-β^+^ and IL-10^+^ T cells. Secretion of all three of these cytokines are implicated the generation of immunotolerance and inhibition of diabetes in NOD mice.[5,6,44] The effect of TA1 was not limited to lymphocyte populations as the miRNA-treated animals also exhibited significant increases in CD11c^+^ dendritic cells (DC). With regards to the CD11c^+^ DC, non-diabetic control and TA1 NOD mice had significantly elevated (p<0.001) levels of these tolerogenic cells relative to the diabetic mice.

The immunomodulatory effect of TA1 was not localized to the pancreatic lymphoid microenvironment. Analyses of immune cell subsets present in the spleen and brachial lymph node of saline and TA1 treated NOD mice similarly demonstrated dramatic changes in the Teff (**Fig 6A**) and tolerogenic (**Fig 6B**) T cell subsets. As in the pancreas, TA1 significantly decreased Teff populations while increasing tolerogenic subsets in both diabetic and non-diabetic NOD mice thus dramatically increasing the Treg:Teff ratio. Moreover, normoglycemic mice exhibited significantly lower levels of the pro-inflammatory Teff cells in both lymphoid tissues. The systemic reorientation of the immune system would argue for the potential utility of the TA1-miRNA therapeutic in other autoimmune diseases.

Much like the complex pattern of miRNA that is necessary for modulating the immune response, a complex interplay of effector and tolerogenic immune cells is also of crucial import in determining if a NOD mouse will develop diabetes. Changes in any number of components of the diabetogenic immune response, including either Teff or tolerogeneic cell populations, may enhance or attenuate progression to overt pathology and diabetes. This differential immunological complexity is noted in **Figs 4**–**6** of the saline and TA1 treated NOD mice that either developed or failed to develop diabetes. However, as shown in **Table 1,** patterns do exist. Assessing the aggregate ratio of effector (E) to tolerogenic (T) cells in the saline treated animals (32.7) clearly demonstrates that a highly proinflammatory environment exists in comparison to the TA1 treated animals (ratio of 3.7). This proinflammatory state arises consequent to both an increase in effector T cells (aggregate increase of 9.8) coupled with an aggregate decrease ratio (0.3) of tolerogenic cells in the diabetic mice relative to non-diabetic mice. Moreover, in trying to elucidate the relative contribution of specific effector cells in disease pathogenesis, **Table 1** clearly demonstrates that Th17^+^ and CD40^+^ cells exhibit a significantly greater presence in diabetic saline treated animals than do Th1 lineages. The efficacy of TA1 in down regulating the proinflammatory state of the pancreatic lymph node can also be seen in **Table 1**. As shown, the aggregate ratio (diabetic:non-diabetic) of effector cells drops from 9.8 to 2.6 (saline and TA1, respectively). Similarly TA1 therapy significantly increased the tolerogenic cell populations relative to the saline treated animals. However, despite the dramatic decrease in the proinflammatory populations and increase in tolerogenic cells, TA1 therapy did not completely prevent T1D, thus reflecting the complexity of disease pathogenesis.

**Table 1:** Ratio of Pancreatic Lymph Node Immune Cells of Saline and TA1 treated Mice: Diabetic/Non-Diabetic Mice. Derived from **Fig 5**.

Cell Populations	Ratio: Diabetic to Non-Diabetic		Ratio: Saline to TA1
	Saline	TA1	
**Effector Cells (E)**			
IFN-γ^+^	2.5	3.7	0.7
Th17^+^	24.0	0.9	27.0
TNF-β^+^	3.2	5.2	0.6
IL-2^+^	3.0	2.3	1.3
CD40^+^	16.2	1.1	14.6
**Mean Aggregate E**	**9.8**	**2.6**	**3.8**
**Tolerogenic Cells (T)**			
Foxp3^+^	0.5	0.9	0.6
IL-4^+^	0.3	0.5	0.6
IL-10^+^	0.1	0.6	0.2
TGF-β^+^	0.2	0.4	0.6
CD11c^+^	0.2	0.9	0.2
**Mean Aggregate T**	**0.3**	**0.7**	**0.4**
***Mean Ratio E*:*T***	***32*.*7***	***3*.*7***	

### TA1 efficacy in the human context

**Fig 7:**
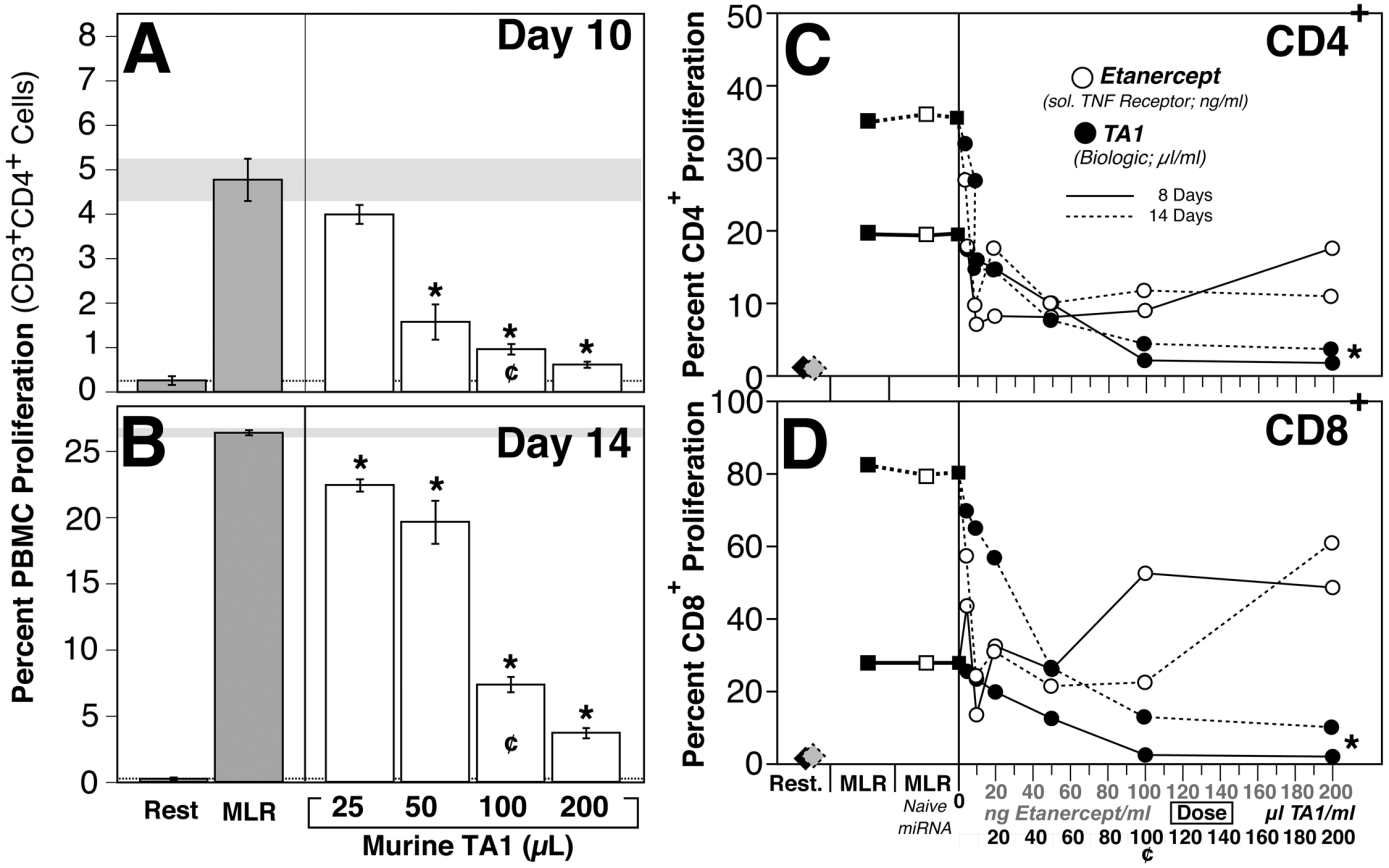
TA1 demonstrates 'drug-like' dosing, efficacy and, consequent to evolutionary conservation, is biologically functional across species. **Panels A-B:** Treatment of human PBMC with murine-derived TA1 inhibits allorecognition in a human PBMC mixed lymphocyte reaction. The horizontal dashed line represents the mean value for resting cells while the horizontal grey bar represents the mean ± SEM for the control MLR value. **Panels C-D:** Both etanercept and TA1 exhibit dose dependent inhibition of PBMC proliferation. Relative to etanercept, TA1 shows equivalent (CD4+; Panel C) or better (CD8+; Panel D) inhibition of human PBMC proliferation after 8 and 14 days of culture. (*) denotes significant (p <0.001 or greater) reduction in proliferation relative to the control MLR. (¢) denotes the concentration used for the *in vivo* murine normal (Figs 1 and 2) and NOD (Figs 3–6) studies. Data shown represents the mean ± SEM of 3 experiments.

While a large number of therapeutic approaches have shown variable degree of success in the NOD mouse, these approaches have not always been found to be effective in the human context.[45] Importantly, miRNA are highly conserved in mammals suggesting a commonality of regulation.[22] To investigate whether the murine-derived TA1 was efficacious in preventing immune recognition in the human context, human mixed lymphocyte reactions (MLR) were performed. As demonstrated in **Fig 7A and 7B**, the murine TA1 exerted a dose-dependent decrease in allorecognition using a two-way MLR model. Indeed at the highest tested dosing (200 μl) of murine-derived TA1, an ~85% reduction in proliferation of human PBMC was noted. Moreover, TA1 demonstrated efficacy in the human context equivalent to that of etanercept (Enbrel^®^; a soluble TNF receptor) a drug currently used in the clinical treatment of autoimmune diseases. As shown in **Fig 7C and 7D**, TA1 resulted in the dose dependent inhibition of alloproliferation of both human CD4^+^ and CD8^+^ PBMC in a MLR. Indeed, relative to etanercept, TA1 demonstrated a significantly improved anti-proliferative effect on cytotoxic CD8^+^ T cells. Importantly, this effect was not due to cytotoxicity of the human PBMC as the murine sourced TA1 did not affect PBMC viability as measured by propidium iodide and 7-AAD staining (data not shown).

## Discussion

Autoimmune destruction of the insulin producing ß cells in T1D is mediated by a T cell-dependent inflammatory disease process characterized by a decrease in the Treg:Teff ratio. The loss of immunological balance most commonly arises from both a decrease in protective regulatory subsets and a concomitant increase in Teff cells. However, current therapeutics exhibit only moderate success in arresting disease progression, typically target only Teff subsets, are generally transient in nature and exert unwanted toxicity to other tissues. A superior approach would be to fundamentally alter the underlying disease state by simultaneously increasing Treg cells while decreasing Teff cells via normal bioregulatory pathways.

Biologics have become a central focus of much of this research. These immunodulatory approaches include cytokine adsorption (*e*.*g*., Etanercept, a soluble TNF-α receptor), exogenous tolerogenic cytokine supplementation, and targeted (typically blocking) monoclonal antibodies.[17–19] However, these approaches do not effectively address the fundamental bioregulatory pathways within the animal that, in a disease state, continue to decrease the Treg cell population. Hence, the global effect of these biologics on the Treg:Teff ratio is often minimal. To successfully alter the Treg:Teff ratio requires a more fundamental bioregulatory approach.

As demonstrated in this study TA1, a miRNA-based therapeutic, directly targets the Treg:Teff ratio yielding a systemic pro-tolerogeneic state both *in vivo* (mouse) and *in vitro* (human and mouse). In immunocompetent mice, TA1 treatment resulted in an increased Treg:Teff ratio consequent to the upregulation of regulatory T cell subsets (*e*.*g*., Tregs and IL10^+^) and the prevention of an inflammatory Teff response upon challenge with allogeneic splenocytes (**Figs 1 and 2**). This multivalent, broad-spectrum, immunomodulatory effect of TA1 was mediated by miRNA as shown by the loss of immunomodulatory activity upon RNase A treatment of either conditioned plasma or the TA1 preparation (**Figs 1 and 2**). More interestingly, TA1 functioned effectively *in vivo* in an autoimmune disease model. In NOD mice the Treg:Teff ratio decreases with age, disease progression and overt hyperglycemia (**Fig 3A**). A low Treg:Teff ratio was associated with an early onset of diabetes while an elevated Treg:Teff ratios were associated with either delayed onset or maintenance of normoglycemia.

Of note, Th17 cells were chosen as the Teff surrogate marker consequent to our findings that these cells were highly expressed in the pancreas of diabetic animals and were virtually absent in non-diabetic mice (**Figs 4 and 5, Table 1**). Furthermore, Th17 cells are potent pro-inflammatory cells and exhibit an inverse relationship to Treg production.[46–49] However, similar findings are observed if Th1 (IFN-γ^+^) cells were used as the surrogate for Teff cells. The Treg:Th1 ratios for non-diabetic and diabetic control NOD mice were 35.8 and 7.7, respectively; a 4.6 fold decrease. In contrast, the Treg:Th1 ratios for TA1-treated non-diabetic and diabetic mice were 147 and 110, respectively; a minimal 1.3 fold change. The observation that TA1 decreased the Th1 (IFN-γ^+^) populations to near baseline levels (**Fig 5A**) in both diabetic and non-diabetic mice suggests that other Teff subsets (*e*.*g*., Th17), or of equal likelihood, a defect in the regulatory subpopulations (**Fig 5B**) may be more critical in disease onset and progression. Indeed, as shown by the lymphoid microenvironment, diabetes is multifactorial and unlikely to be solely mediated by a single T cell subset.

The effect of TA1 on other cell types may also be of importance. As shown, TA1 upregulated both CD25^+^ cells and CD69^+^ cells in immunocompetent mice *in vivo*. In diabetic NOD mice, DC were significantly (p<0.01) upregulated by TA1 relative to saline treated animals. Moreover, in the non-diabetic animals (saline and TA1), DC were greatly elevated (p<0.01) relative to the diabetic animals and showed no difference between the saline and TA1 treated animals. Of note, previous studies have implicated the presence of tolerogenic CD11c^+^ DC in preventing T1D disease progression in NOD mice. [50–52] In aggregate, these data clearly demonstrate that TA1 significantly reset the pancreatic lymphoid microenvironment towards a non-inflammatory state that yielded a significant reduction in disease progression towards overt diabetes. Also of significance, diabetic mice (both saline and TA1-treated) tended to have significantly lower levels of all tolerogenic subsets than non-diabetic (control or TA1) mice thus further exacerbating the decrease in the Treg:Teff ratio.

The potential utility of this immunomodulatory approach is further supported by the timing of the TA1 administration. As noted, TA1 was administered at 7 weeks of age, well beyond the optimal window for therapeutic intervention (3–4 weeks of age).[2] Despite the onset of the disease process, TA1 was still able to significantly altered the kinetics of change in the Treg:Teff ratio and decrease the overall incidence of T1D. The TA1-mediated increase in the Treg:Teff ratio arose from the decrease of *multiple Teff subsets* with a corresponding increase in *multiple regulatory/tolerogenic T cell subsets* in lymphoid tissues (**Figs 4–6, Table 1**). The increase Treg:Teff ratio was correlated with a reduction of leukocyte infiltration of the pancreatic lymph nodes (**Fig 3B**). Finally, and perhaps not too surprisingly due to their evolutionary conservation, the murine-derived TA1-miRNA was also highly effective in modulating human leukocyte allorecognition *in vitro* (**Fig 7A and 7B**). Similar to traditional therapeutic agents, the TA1-miRNA product demonstrated a dose response curve in *in vitro* human MLR studies (**Fig 7C and 7D**).

The miRNA species complexity of TA1 was done purposefully. Despite the inherent reductionist nature of science and clinical medicine, an increase or decrease of a single miRNA, while potentially useful as a biomarker, is unlikely to exert a potent therapeutic effect. Indeed, based on the known biology and promiscuity of individual miRNA species and the genes they regulate, *‘patterns of expression’* (encompassing static, increased and decreased levels of multiple miRNA species) may be crucial in the development of effective miRNA-based therapeutics. It was this functional *‘pattern of miRNA expression’* that we chose to biologically manufacture in an attempt to achieve maximal immunomodulatory functionality. As demonstrated in both immunologically normal and NOD mice, and *in vitro* human studies, the relatively complex TA1 pattern of miRNA expression (**Fig 1D**) effectively induced a tolerogenic state. From a drug perspective, it may be useful to think of TA1 as being analogous to clinically used human intravenous immunoglobulin (IVIG; pooled human IgG prepared from several thousand donors) in that TA1 is a ‘complex’ preparation consisting of a broad range of miRNA expressed with variable abundance relative to naïve miRNA.

Can miRNA therapeutics be stable and reproducible pharmacologic agents? In contrast to highly labile mRNA, miRNA are relatively stable. The TA1 preparation described withstood repeated freeze-thaw conditions, exhibited dose dependent activity and showed equivalent efficacy with current, clinically used, immunomodulatory drugs (**Fig 7**). While the direct mechanism(s) and tissue site(s) by which TA1 modulates the immune response are not fully understood, evidence to date suggests that multiple lymphoid organs, including the thymus, are targeted by the exogenous miRNA. Analysis of the putative functions (see **S1 Table**) described for the miRNA species identified is suggestive of potential cellular mechanisms with multiple species being implicated in cellular differentiation and proliferation. Indeed, miR-206 expression is inversely correlated with Th17 proliferation.[53] However, as with any 'drug', it is also important to note that TA1 may also pose potential toxicity issues due to its potent immunomodulatory effect. Indeed, several miRNA identified in **Fig 1D** have been implicated in, or more likely as biomarkers of, carcinogenesis. Chronic immunosuppression using conventional therapeutics is known to lead to increased risk of cancer and an inability to respond appropriately to exogenous pathogens. It is possible, even probable, that a comparable risk exists for TA1. Hence further attention to these potential TA1 risks, relative to existing immunosuppressive agents, will be necessary.

As demonstrated, the TA1-miRNA systemically reoriented the immune system of normal and NOD mouse towards a tolerogenic state by increasing the Treg:Teff ratio. In NOD mice this increase inhibited insulitis (*i*.*e*., improved histology) and T1D incidence. Indeed, as shown, TA1 exerts a broad-spectrum effect: upregulating regulatory subsets while simultaneously downregulating Teff subsets yielding a significant increase in the Treg:Teff ratio in multiple lymphoid tissues. Ongoing studies are examining the effect of different dosing regimes, timing of drug administration (pre- and post onset of disease) and comparing TA1 to current immunosuppressive drugs. The findings of this study provide significant new insights into the potential utility of miRNA-based therapeutics to attenuate not only T1D but other autoimmune diseases as well. The same miRNA-approach may prove valuable in preventing allorejection of donor tissues as demonstrated by the ability of the TA1-miRNA to inhibit allorecognition in a human MLR model.

## Supporting Information

S1 Fig TA1 therapy decreased multiple Teff cell subpopulations (relative to control mice) in the pancreatic lymph node of both diabetic and non-diabetic NOD mice. Shown are representative flow cytometric data for Saline-Diabetic, TA1-Diabetic, Saline-NonDiabetic and TA1-NonDiabetic mice as presented in **Fig 5**. The saline-treated control NOD mouse group consisted of 16 animals while the TA1 treated cohort consisted of 15 animals. To best reflect the *in situ* leukocyte subpopulations mediating the autoimmune disease pathology, no exogenous stimulation of the isolated cells was done prior to flow cytometric analysis.
(TIF)

S2 Fig TA1 therapy increased multiple toloerogenic cell supopulations (relative to control mice) in the pancreatic lymph node of both diabetic and non-diabetic NOD mice. Shown are representative flow cytometric data for Saline-Diabetic, TA1-Diabetic, Saline-NonDiabetic and TA1-NonDiabetic mice as presented in **Fig 5**. The saline-treated control NOD mouse group consisted of 16 animals while the TA1 treated cohort consisted of 15 animals. To best reflect the *in situ* leukocyte subpopulations mediating the autoimmune disease pathology, no exogenous stimulation of the isolated cells was done prior to flow cytometric analysis.
(TIF)

S1 Table Putative Function(s) of Identified TA1 miRNA Species. U = Upregulated; D = Downregulated.
(DOCX)
