# Supplementary figures and images for: Inhibition of Autoimmune Diabetes in NOD Mice by miRNA Therapy
Source: PLoS One. 2015 Dec 16;10(12):e0145179. doi: 10.1371/journal.pone.0145179 (PMC4692265; doi:10.1371/journal.pone.0145179)

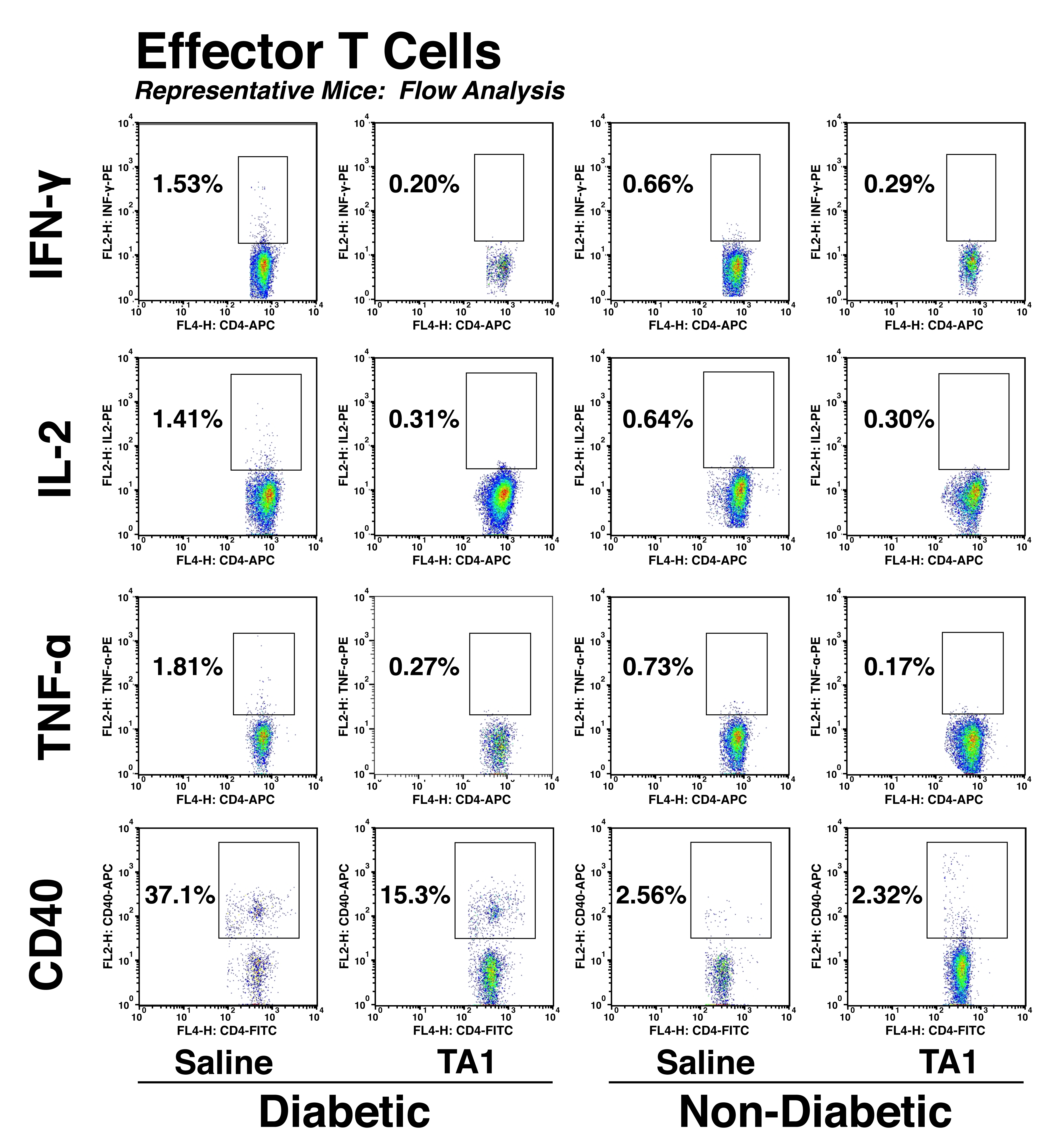

Supplement: S1 Fig — Shown are representative flow cytometric data for Saline-Diabetic, TA1-Diabetic, Saline-NonDiabetic and TA1-NonDiabetic mice as presented in Fig 5. The saline-treated control NOD mouse group consisted of 16 animals while the TA1 treated cohort consisted of 15 animals. To best reflect the in situ leukocyte subpopulations mediating the autoimmune disease pathology, no exogenous stimulation of the isolated cells was done prior to flow cytometric analysis. (TIF) [file pone.0145179.s001.tif]

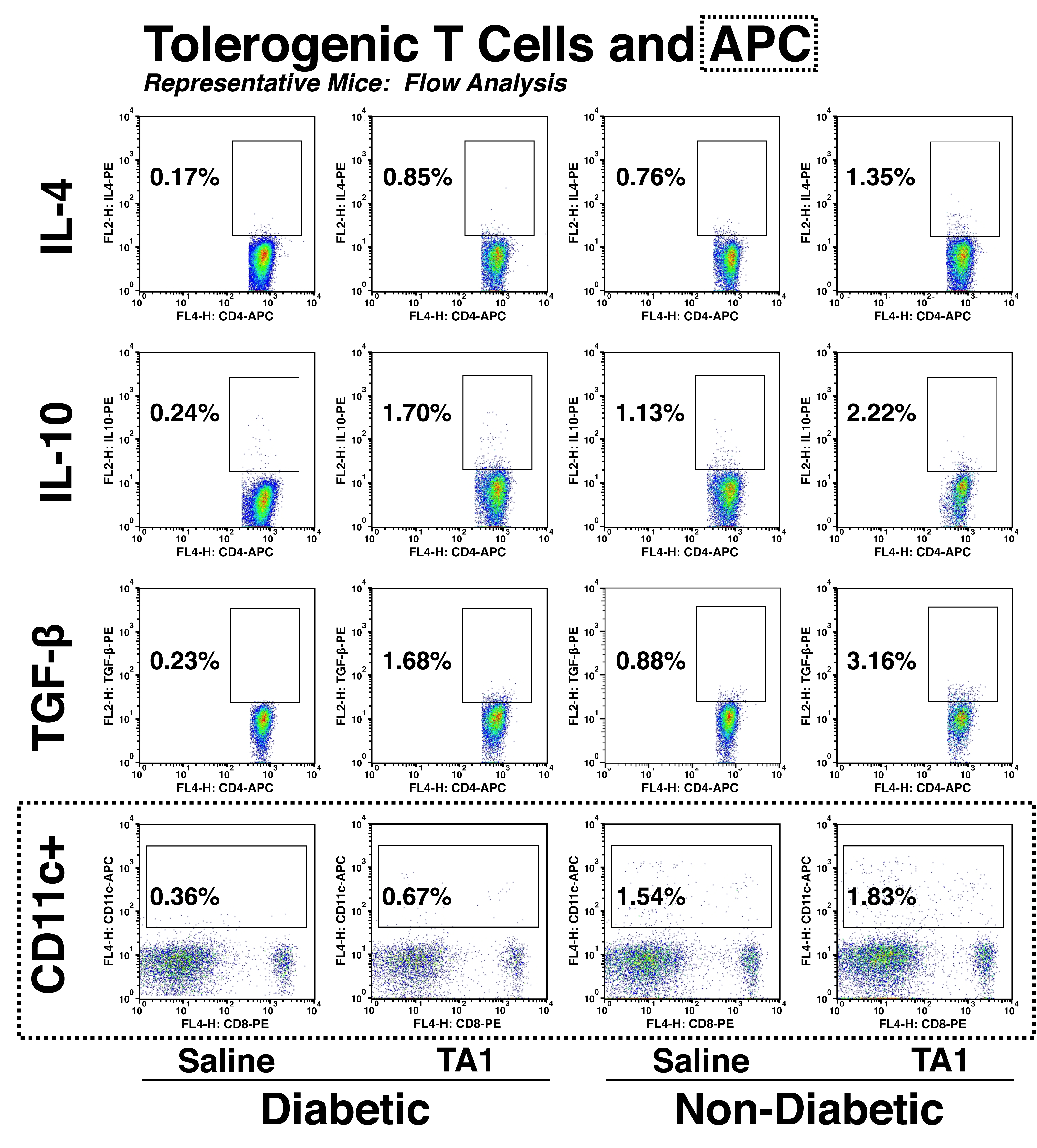

Supplement: S2 Fig — Shown are representative flow cytometric data for Saline-Diabetic, TA1-Diabetic, Saline-NonDiabetic and TA1-NonDiabetic mice as presented in Fig 5. The saline-treated control NOD mouse group consisted of 16 animals while the TA1 treated cohort consisted of 15 animals. To best reflect the in situ leukocyte subpopulations mediating the autoimmune disease pathology, no exogenous stimulation of the isolated cells was done prior to flow cytometric analysis. (TIF) [file pone.0145179.s002.tif]
