# Supplementary material for: Inhibition of Autoimmune Diabetes in NOD Mice by miRNA Therapy
Source: PLoS One. 2015 Dec 16;10(12):e0145179. doi: 10.1371/journal.pone.0145179 (PMC4692265; doi:10.1371/journal.pone.0145179)
Supplement: S1 Table — U = Upregulated; D = Downregulated. (DOCX) [file pone.0145179.s003.docx]

**Supplemental Table 1.**  Putative Function(s) of Identified TA1 miRNA Species. U = Upregulated; D = Downregulated

| **miRNA** | U/D | **Putative Function(s) -** *Often in the Context of Biomarkers* |
| --- | --- | --- |
| **miR-214-3p** | U︎ | miR-214 is predicted to target two activating protein 2 transcription factors, bringing about downstream effects on a number of genes regulating vital cell cycle processes, such as apoptosis, proliferation and angiogenesis.[1] Upregulation of miR-214 in Hela cells reduced cell growth.[2] Elevated expression in pancreatic cancer may inhibit chemotherapy effectiveness.[3] |
| **miR-149-5p** | U︎ | Tumor suppressor activity associated with the regulation of apoptosis.[4] Expression of miR-149 is inversely associated with inflammation. miR-149 has regulatory effects on TNF-α, IL-1ß and IL-6.[5] |
| **miR-147a** | U | Implicated along with **miR-155** and **miR21** (see this table) in regulation of Toll-like receptors (TLRs). The dysregulation of these miRNAs may be involved in inflammatory diseases and cancers.[6] |
| **miR-302a-3p** | U︎ | Studies have shown that miR-302 is able to reprogram human cancer cells to human embryonic stem cell (hESC)–like pluripotent cells with a slow cell cycle rate and dormant cell-like morphology.[7,8] Inhibits CDK2 and CDK4/6 cell cycle pathways.[9] |
| **miR-206** | U︎ | Inverse realtionship beetween miR-206 expression and **Th17 cells** in an inflammatory disease (dermatomyositis). [10] |
| **miR-298** | U︎ | miR-298-5p and miR-296-3p are causally involved in the higher resistance of mammalian pancreatic α cells to cytokine-induced apoptosis. [11] |
| **miR-34a-5p** | U︎ | miR-34a is a part of the p53 tumor suppressor network. It is hypothesized that miR-34 dysregulation is involved in the development of inflammation and some cancers.[12,13] |
| **miR-135b-5p** | U︎ | miR-135b is reported to be a is an oncogenic microRNA that enhances cancer cell invasive and migratory abilities in vitro and promotes cancer metastasis in vivo.[14] Other studies demonstrate that miR135b is involved in the regulation of inflammation. miR-135b expression in inflammation is regulated by IL-1R1 in a regulatory feedback mechanism to resolve inflammation.[15] |
| **miR-9-5p** | U︎ | miR-9 induced by TLR4 activation as well as TLR2 and TLR7/8 agonists and by the proinflammatory cytokines (TNF-α and IL-1β, but not by IFN-γ). [16] It has been shown however that miR-9 expression is reduced in some cancers.[17] |
| **miR-155-5p** | U︎ | miR-155 expression inhibits malignant growth *in vivo*.[18] *see also* **miR-147a.** |
| **miR-132-3p** | U︎ | miR-132 is extensively involved in the modulation inflammation. One putative target being p300. Down regulation of p300 inhibits expression of IFN-β, ISG15, IL-1β and IL6.[19] In autoimmune rheumatoid arthritis low plasma levels of miR-132 are observed suggesting a dysregulation of inflammation.[20] |
| **let-7e-5p**  **let-7a-5p** | U | The lethal-7 (let-7) gene and miRNA were one of the first two known miRNAs (the other one is lin-4) in C. elegans and humans.[21] Evidence suggests that a major function of let-7 genes may be to promote terminal differentiation and apoptosis in development and to act as a 'cancer suppressor'. Let-7 is implicated in the regulation of IL-6, IL-10, IL-13 and a negative regulator of TLR4.[22] |
| **miR-27b-3p**  **miR-27a-3p** | U︎ | miR-27a and -27b are implicated in cellular differentiation and are activators of the Wnt signalling pathway.[23] miR-27a has been identified as one of three miRNAS (along with miR-96 and miR-182) which directly target FOXO1 and regulate its endogenous expression. Suppression of miR-27a results in a FOXO1 protein increase and a consequent cell number decrease.[24] The FOXO transcription factor family--which is central to the integration of growth factor signalling, oxidative stress and inflammation may modulate the magnitude of an immune response. FOXO transcription factors may guide T cell differentiation and function in a context-driven manner.[25] |
| **miR-21-5p** | U︎ | A number of targets for miR-21 have been experimentally validated and most of them are tumor suppressors. miR-21 may work in conjunction with miR-34a.[26] *see also* **miR-34a.** |
| **miR-363-3p** | D︎ | miR-363 may regulate endothelial cell properties and their communication with hematopoietic precursor cells.[27] Overexpression of miR-363 suppresses the tumourigenicity of colorectal cancer cells.[28] |
| **miR-203a** | D | miR-203 has been found overexpressed in pancreatic adenocarcinoma and is correlated with poor prognosis. The expression of this miR-203 is induced by various cytokines, including IL6 and IFN-γ.[29] |
| **miR-183-5p** | D | The miR-183/-96/-182 polycistronic miRNA cluster is up-regulated in most breast cancers and increases cell proliferation and migration.[30] miR-183 is negatively regulated by transcription factor GATA3.[31] |

**References Cited:**

1. Bar-Eli M. Searching for the ‘melano-miRs’: miR-214 drives melanoma metastasis. EMBO J. 2011;30: 1880-1881.

2. Yang Z, Chen S, Luan X, Li Y, Liu M, Li X et al. MicroRNA-214 is aberrantly expressed in cervical cancers and inhibits the growth of HeLa cells. IUBMB Life. 2009;61: 1075-1082.

3. Zhang XJ, Ye H, Zeng CW, He B, Zhang H, Chen YQ. Dysregulation of miR-15a and miR-214 in human pancreatic cancer. J Hematol Oncol. 2010;3: 46.

4. Lin RJ, Lin YC, Yu AL. miR-149* induces apoptosis by inhibiting Akt1 and E2F1 in human cancer cells. Mol Carcinog. 2010;49: 719-727.

5. Santini P, Politi L, Vedova PD, Scandurra R, Scotto d’Abusco A. The inflammatory circuitry of miR-149 as a pathological mechanism in osteoarthritis. Rheumatol Int. 2014;34: 711-716.

6. Quinn SR, O’Neill LA. A trio of microRNAs that control Toll-like receptor signalling. Int Immunol. 2011;23: 421-425.

7. Lin SL, Chang DC, Chang-Lin S, Lin CH, Wu DT, Chen DT et al. Mir-302 reprograms human skin cancer cells into a pluripotent ES-cell-like state. RNA. 2008;14: 2115-2124.

8. Barroso-del Jesus A, Lucena-Aguilar G, Menendez P. The miR-302-367 cluster as a potential stemness regulator in ESCs. Cell Cycle. 2009;8: 394-398.

9. Lin SL, Chang DC, Ying SY, Leu D, Wu DT. MicroRNA miR-302 inhibits the tumorigenecity of human pluripotent stem cells by coordinate suppression of the CDK2 and CDK4/6 cell cycle pathways. Cancer Res. 2010;70: 9473-9482.

10. Tang X, Tian X, Zhang Y, Wu W, Tian J, Rui K et al. Correlation between the frequency of Th17 cell and the expression of microRNA-206 in patients with dermatomyositis. Clin Dev Immunol. 2013;2013: 345347.

11. Barbagallo D, Piro S, Condorelli AG, Mascali LG, Urbano F, Parrinello N et al. miR-296-3p, miR-298-5p and their downstream networks are causally involved in the higher resistance of mammalian pancreatic alpha cells to cytokine-induced apoptosis as compared to beta cells. BMC Genomics. 2013;14: 62.

12. Chen QR, Yu LR, Tsang P, Wei JS, Song YK, Cheuk A et al. Systematic proteome analysis identifies transcription factor YY1 as a direct target of miR-34a. J Proteome Res. 2011;10: 479-487.

13. He L, He X, Lim LP, de Stanchina E, Xuan Z, Liang Y et al. A microRNA component of the p53 tumour suppressor network. Nature. 2007;447: 1130-1134.

14. Lin CW, Chang YL, Chang YC, Lin JC, Chen CC, Pan SH et al. MicroRNA-135b promotes lung cancer metastasis by regulating multiple targets in the Hippo pathway and LZTS1. Nat Commun. 2013;4: 1877.

15. Halappanavar S, Nikota J, Wu D, Williams A, Yauk CL, Stampfli M. IL-1 receptor regulates microRNA-135b expression in a negative feedback mechanism during cigarette smoke-induced inflammation. J Immunol. 2013;190: 3679-3686.

16. Bazzoni F, Rossato M, Fabbri M, Gaudiosi D, Mirolo M, Mori L et al. Induction and regulatory function of miR-9 in human monocytes and neutrophils exposed to proinflammatory signals. Proc Natl Acad Sci U S A. 2009;106: 5282-5287.

17. Lehmann U, Hasemeier B, Christgen M, Muller M, Romermann D, Langer F et al. Epigenetic inactivation of microRNA gene hsa-mir-9-1 in human breast cancer. J Pathol. 2008;214: 17-24.

18. Babar IA, Cheng CJ, Booth CJ, Liang X, Weidhaas JB, Saltzman WM et al. Nanoparticle-based therapy in an in vivo microRNA-155 (miR-155)-dependent mouse model of lymphoma. Proc Natl Acad Sci U S A. 2012;109: E1695-704.

19. Lagos D, Pollara G, Henderson S, Gratrix F, Fabani M, Milne RS et al. miR-132 regulates antiviral innate immunity through suppression of the p300 transcriptional co-activator. Nat Cell Biol. 2010;12: 513-519.

20. Murata K, Yoshitomi H, Tanida S, Ishikawa M, Nishitani K, Ito H et al. Plasma and synovial fluid microRNAs as potential biomarkers of rheumatoid arthritis and osteoarthritis. Arthritis Res Ther. 2010;12: R86.

21. Pasquinelli AE, Reinhart BJ, Slack F, Martindale MQ, Kuroda MI, Maller B et al. Conservation of the sequence and temporal expression of let-7 heterochronic regulatory RNA. Nature. 2000;408: 86-89.

22. Barh D, Malhotra R, Ravi B, Sindhurani P. MicroRNA let-7: an emerging next-generation cancer therapeutic. Curr Oncol. 2010;17: 70-80.

23. Wang T, Xu Z. miR-27 promotes osteoblast differentiation by modulating Wnt signaling. Biochem Biophys Res Commun. 2010;402: 186-189.

24. Guttilla IK, White BA. Coordinate regulation of FOXO1 by miR-27a, miR-96, and miR-182 in breast cancer cells. J Biol Chem. 2009;284: 23204-23216.

25. Hedrick SM, Hess Michelini R, Doedens AL, Goldrath AW, Stone EL. FOXO transcription factors throughout T cell biology. Nat Rev Immunol. 2012;12: 649-661.

26. Hashimi ST, Fulcher JA, Chang MH, Gov L, Wang S, Lee B. MicroRNA profiling identifies miR-34a and miR-21 and their target genes JAG1 and WNT1 in the coordinate regulation of dendritic cell differentiation. Blood. 2009;114: 404-414.

27. Costa A, Afonso J, Osorio C, Gomes AL, Caiado F, Valente J et al. miR-363-5p regulates endothelial cell properties and their communication with hematopoietic precursor cells. J Hematol Oncol. 2013;6: 87.

28. Tsuji S, Kawasaki Y, Furukawa S, Taniue K, Hayashi T, Okuno M et al. The miR-363-GATA6-Lgr5 pathway is critical for colorectal tumourigenesis. Nat Commun. 2014;5: 3150.

29. Greither T, Grochola LF, Udelnow A, Lautenschlager C, Wurl P, Taubert H. Elevated expression of microRNAs 155, 203, 210 and 222 in pancreatic tumors is associated with poorer survival. Int J Cancer. 2010;126: 73-80.

30. Li P, Sheng C, Huang L, Zhang H, Huang L, Cheng Z et al. MiR-183/-96/-182 cluster is up-regulated in most breast cancers and increases cell proliferation and migration. Breast Cancer Res. 2014;16: 473.

31. Chen C, Xiang H, Peng YL, Peng J, Jiang SW. Mature miR-183, negatively regulated by transcription factor GATA3, promotes 3T3-L1 adipogenesis through inhibition of the canonical Wnt/beta-catenin signaling pathway by targeting LRP6. Cell Signal. 2014;26: 1155-1165.
